# Crystal structure, Hirshfeld surface analysis, inter­action energy, and DFT studies of cholesteryl hepta­noate

**DOI:** 10.1107/S2056989021005661

**Published:** 2021-06-04

**Authors:** Nurcan Akduran, Tuncay Karakurt, Tuncer Hökelek

**Affiliations:** a Department of Metallurgical and Materials Engineering, Faculty of Technology, Selçuk University, 42130 Selçuklu, Konya, Turkey; b Department of Chemical Engineering, Faculty of Engineering & Architecture, Kırşehir Ahi Evran University, 40100, Kırşehir, Turkey; c Department of Physics, Hacettepe University, 06800 Beytepe, Ankara, Turkey

**Keywords:** crystal structure, cholester­yl, cholesterol

## Abstract

The title compound, C_34_H_58_O_2_, consists of cholesteryl and hepta­noate units, in which the six-membered rings adopt chair and twisted-boat conformations while the five-membered ring adopts an envelope conformation. In the crystal, the mol­ecules are aligned along the *a*-axis direction and stacked along the *b*-axis direction. The Hirshfeld surface analysis of the crystal structure indicates that the most important contributions for the crystal packing are from H⋯H (92.4%) and H⋯O/O⋯H (6.1%) inter­actions. van der Waals inter­actions are the dominant inter­actions in the crystal packing. Density functional theory (DFT) optimized structures at the B3LYP/ 6–31 G(*d*) level are compared with the experimentally determined mol­ecular structure in the solid state. The HOMO–LUMO behaviour was elucidated to determine the energy gap, and the mol­ecular electrostatic potential (MEP) of the compound was investigated.

## Abstract 2

The title compound consists of cholesteryl and hepta­noate units, in which the six-membered rings adopt chair and twisted-boat conformations, while the five-membered ring adopts an envelope conformation. In the crystal, the mol­ecules are aligned along the *a*-axis direction and stacked along the *b*-axis direction.

## Chemical context   

Cholesterol is an important constituent of cell membranes with a rigid ring system and a short branched hydro­carbon tail. It modulates membrane fluidity over the range of physiological temperatures and also reduces the permeability of the plasma membrane to protons and sodium ions. In the liver, it is converted to bile, which is then stored in the gallbladder. It functions in intra­cellular transport, cell signaling and nerve conduction within the cell membrane and is an important precursor in several biochemical pathways within the cells, in the synthesis of vitamin D and steroid hormones, including the adrenal gland hormones cortisol and aldosterone as well as sex hormones progesterone, oestrogens, and testosterone, and their derivatives. Cholesteryl esters are formed between the carboxyl­ate group of a fatty acid and the hydroxyl group of cholesterol and have a lower solubility in water than cholesterol. These esters are also important in many biological mechanisms and numerous experimental investigations have been performed on cholesterol derivatives (Faiman *et al.*, 1976 ▸; Goheen *et al.*, 1977 ▸; Bush *et al.*, 1980 ▸; Di Vizio *et al.*, 2008 ▸; Ikonen, 2008 ▸). Thus, due to the importance of cholesterol and its esters, we report herein the crystallization, the mol­ecular and crystal structures along with the Hirshfeld surface analysis and the inter­action energy and DFT studies of the title compound, (I), whose magnetic properties were previously studied by electron paramagnetic resonance (EPR), (Sayin *et al.*, 2013 ▸).
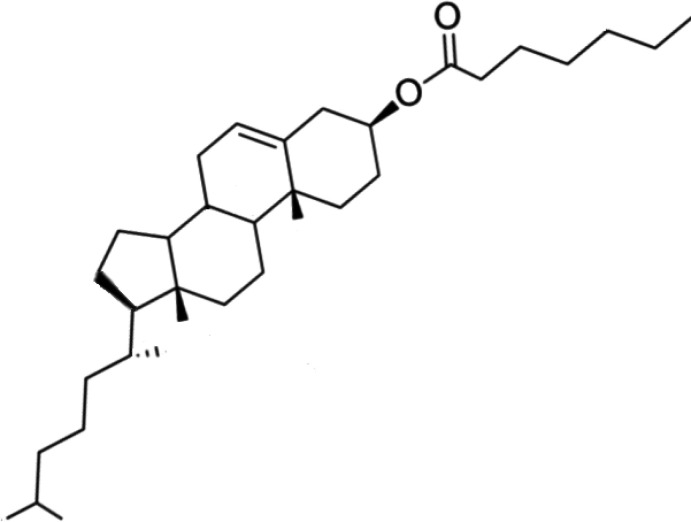


## Structural commentary   

**Figure 1:**
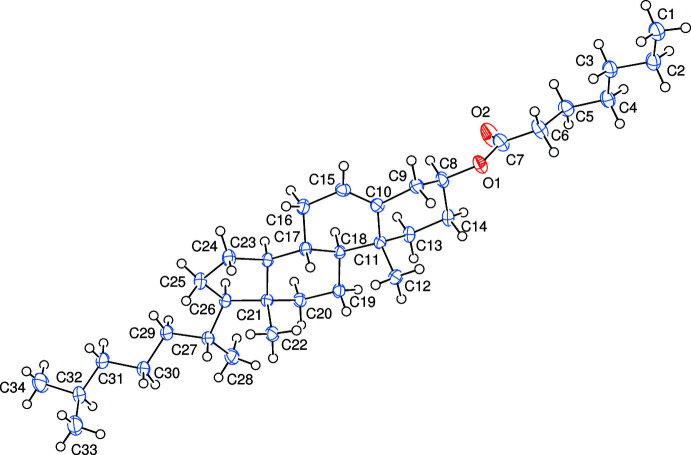
The asymmetric unit of the title compound with the atom-numbering scheme. Displacement ellipsoids are drawn at the 50% probability level.

As shown in Fig. 1 ▸, the title compound, (I), consists of cholesteryl and hepta­noate units. A puckering analysis (Cremer & Pople, 1975 ▸) of the six-membered *A* (C8–C11/C13/C14), *B* (C10/C11/C15–C18), *C* (C17–C21/C23) and the five-membered *D* (C23–C26/C21) rings gave the parameters [*Q*_T_ = 0.5403 (16) Å, θ = 6.86 (18)° and φ = 327.4 (15)°, adopting a chair conformation (for *A*), *Q*_T_ = 0.4839 (15) Å, θ = 129.5 (3)° and φ = 328.2 (2)°, adopting a twisted-boat conformation (for *B*), *Q*_T_ = 0.5646 (15) Å, θ = 6.44 (14)° and φ = 245.1 (14)°, adopting a chair conformation (for *C*) and *q*_2_ = 0.4635 (16) Å and φ = 191.7 (2)°, adopting an envelope conformation, where atom C21 is at the flap position and 0.693 (2) Å away from best plane of the remaining atoms (for *D*)]. The O1—C7 [1.348 (3) Å] and O2—C7 [1.196 (3) Å] bonds in the carboxyl­ate group indicate localized single and double bonds. The O1—C7—O2 [123.8 (2)°] bond angle seems to be increased compared to that present in a free acid [122.2°].

## Supra­molecular features   

**Figure 2:**
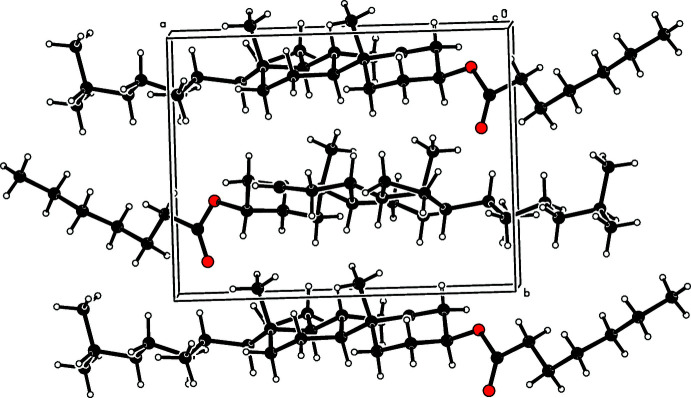
A partial packing diagram viewed down the *c* axis.

In the crystal, the mol­ecules are aligned along the *a*-axis direction and stacked along the *b*-axis direction (Fig. 2 ▸).

## Hirshfeld surface analysis   

**Figure 3:**
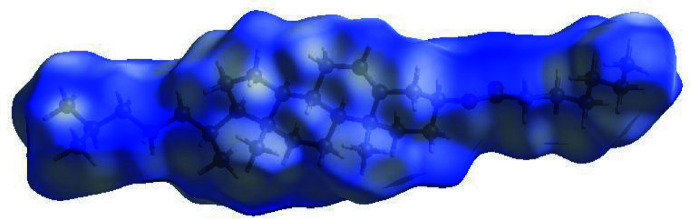
View of the three-dimensional Hirshfeld surface of the title compound plotted over *d*_norm_ in the range of 0.0196 to 1.7047 a.u.

**Figure 4:**
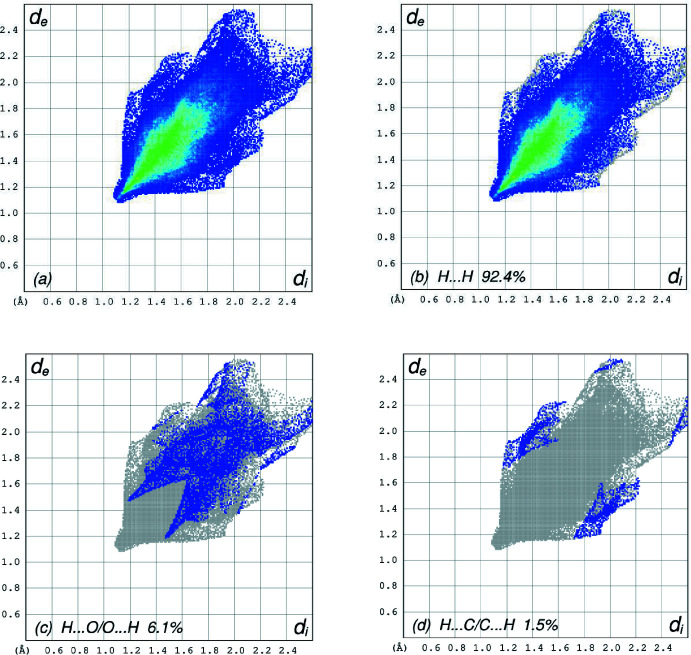
The full two-dimensional fingerprint plots for the title compound, showing (*a*) all inter­actions, and those delineated into (*b*) H⋯H, (*c*) H⋯O/O⋯H and (*d*) H⋯C/C⋯H inter­actions. The *d*_i_ and *d*_e_ values are the closest inter­nal and external distances (in Å) from given points on the Hirshfeld surface contacts.

In order to visualize the inter­molecular inter­actions in the crystal of the title compound, a Hirshfeld surface (HS) analysis (Hirshfeld, 1977 ▸; Spackman & Jayatilaka, 2009 ▸) was carried out by using *Crystal Explorer 17.5* (Turner *et al.*, 2017 ▸). In the HS plotted over *d*_norm_ (Fig. 3 ▸), the white surface indicates contacts with distances equal to the sum of van der Waals radii, and the red and blue colours indicate distances shorter (in close contact) or longer (distinct contact) than the van der Waals radii, respectively (Venkatesan *et al.*, 2016 ▸). The bright-red spots indicate their roles as the respective donors and/or acceptors. The overall two-dimensional fingerprint plot, Fig. 4 ▸ *a*, and those delineated into H⋯H, H⋯O/O⋯H and H⋯C/C⋯H contacts (McKinnon *et al.*, 2007 ▸) are illustrated in Fig. 4 ▸ *b*–*d*, respectively, together with their relative contributions to the Hirshfeld surface. The most important inter­action is H⋯H (Table 1 ▸) contributing 92.4% to the overall crystal packing, which is reflected in Fig. 4 ▸ *b* as widely scattered points of high density due to the large hydrogen content of the mol­ecule with the tip at *d*_e_ = *d*_i_ = 1.11 Å. The pair of spikes in the fingerprint plot delineated into H⋯O/O⋯H contacts (Table 1 ▸) have a symmetrical distribution of points (6.1% contribution, Fig. 4 ▸ *c*) with the tips at *d*_e_ + *d*_i_ = 2.66 Å. In the absence of C—H⋯π inter­actions, the pair of characteristic wings in the fingerprint plot delineated into H⋯C/C⋯H contacts (Table 1 ▸, Fig. 4 ▸ *c*, 1.5% contribution) has the tips at *d*_e_ + *d*_i_ = 2.89 Å.

**Figure 5:**
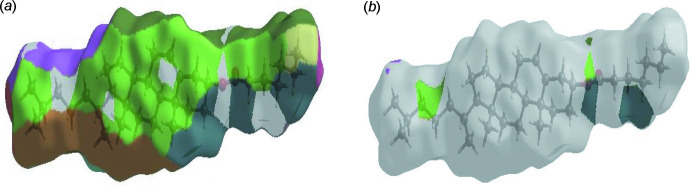
The Hirshfeld surface representations with the function *d*_norm_ plotted onto the surface for (*a*) H⋯H and (*b*) H⋯O/O⋯H inter­actions.

The Hirshfeld surface representations with the function *d*_norm_ plotted onto the surface are shown for the H⋯H and H⋯O/O⋯H inter­actions in Fig. 5 ▸ *a*–*b*, respectively.

The Hirshfeld surface analysis confirms the importance of H-atom contacts in establishing the packing. The large number of H⋯H and H⋯O/O⋯H inter­actions suggest that van der Waals inter­actions play the major role in the crystal packing (Hathwar *et al.*, 2015 ▸).

## Inter­action energy calculations   

The inter­molecular inter­action energies are calculated using the CE–B3LYP/6–31G(d,p) energy model available in *Crystal Explorer 17.5* (Turner *et al.*, 2017 ▸), where a cluster of mol­ecules is generated by applying crystallographic symmetry operations with respect to a selected central mol­ecule within the radius of 3.8 Å by default (Turner *et al.*, 2014 ▸). The total inter­molecular energy (*E*_tot_) is the sum of electrostatic (*E*_ele_), polarization (*E*_pol_), dispersion (*E*_dis_) and exchange-repulsion (*E*_rep_) energies (Turner *et al.*, 2015 ▸) with scale factors of 1.057, 0.740, 0.871 and 0.618, respectively (Mackenzie *et al.*, 2017 ▸). The evaluation of the energies indicates that the stabilizations in the title compound are dominated by the dispersion energy contributions.

## DFT calculations   

**Figure 6:**
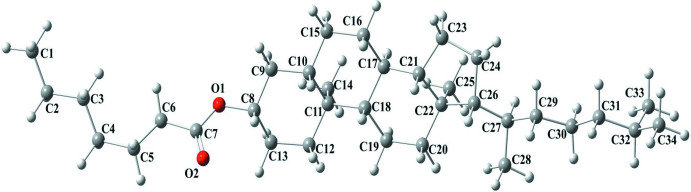
The optimized structure of the title compound, (I).

**Figure 7:**
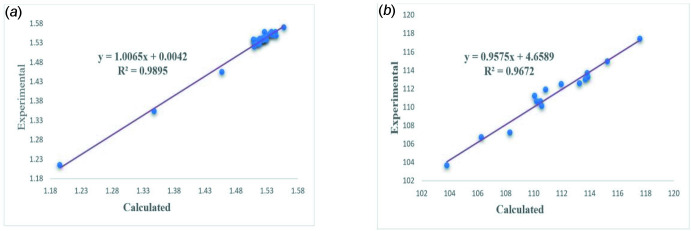
The correlation graphs of the calculated and experimental (*a*) bond lengths and (*b*) bond angles of the title compound, (I).

**Figure 8:**
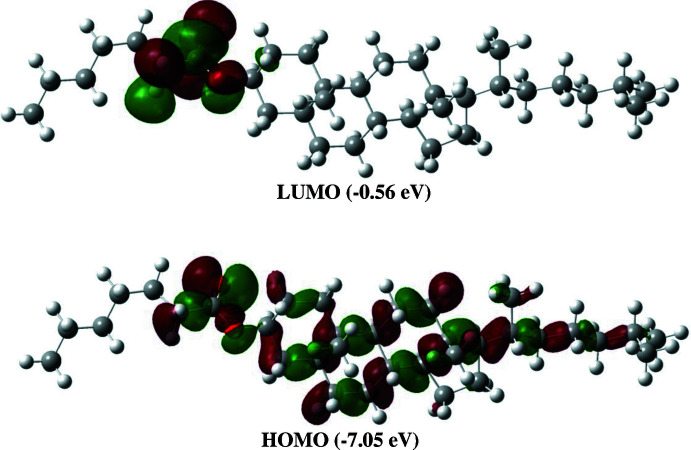
The LUMO and HOMO energies of the title compound, (I).

The optimized structure (Fig. 6 ▸) of the title compound was generated theoretically *via* density functional theory (DFT) using standard B3LYP functional and 6–31 G(d) basis-set calculations (Becke, 1993 ▸) as implemented in *GAUSSIAN 09* (Frisch *et al.*, 2009 ▸). The theoretical and experimental results were in good agreement (Table 2 ▸). As is common in these studies, there are differences between the observed and calculated values because the former pertain to the solid state while the latter are for an isolated mol­ecule in the gas phase. The correlation graphs based on the calculations of the bond lengths and angles for comparison with the experimental results are shown in Fig. 7 ▸ *a* and *b*, respectively. The highest-occupied mol­ecular orbital (HOMO), acting as an electron donor, and the lowest-unoccupied mol­ecular orbital (LUMO), acting as an electron acceptor, are very important parameters for quantum chemistry. When the energy gap is small, the mol­ecule is highly polarizable and has high chemical reactivity and it is characterized as soft. The DFT calculations provide some important information on the reactivity and site selectivity of the mol­ecular framework. *E*_HOMO_ and *E*_LUMO_ clarify the inevitable charge exchange collaboration inside the studied material, electronegativity (χ), hardness (η), potential (μ), electrophilicity (ω) and softness (*σ*) are recorded in Table 3 ▸. The significance of η and *σ* is to evaluate both the reactivity and stability. The HOMO and LUMO energy levels are shown in Fig. 8 ▸. The HOMO is localized in the plane extending over the whole cholesteryl hepta­noate ring, while the LUMO is localized on the oxygens and their surrounding atoms. The energy band gap [Δ*E* = *E*_LUMO_ − *E*_HOMO_] of the mol­ecule is 6.49 eV, and the frontier mol­ecular orbital energies, *E*_HOMO_ and *E*_LUMO_ are −7.05 and −0.56 eV, respectively.

**Figure 9:**
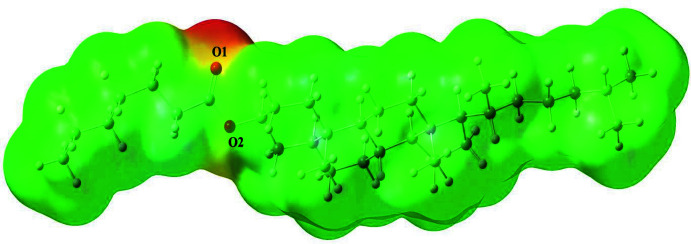
The MEP plot of the title compound, (I).

The mol­ecular electrical potential surfaces or electrostatic potential energy maps illustrate the charge distributions of the mol­ecules in three dimensions, allowing one to visualize variably charged regions of the mol­ecule, which may be used to determine how mol­ecules inter­act with one another. Electrostatic potential maps (MEPs) are invaluable in predicting the behaviour of complex mol­ecules. The MEP of the title compound is shown in Fig. 9 ▸, where the negative electrostatic potential formed around O1 and O2 atoms and positive potential (green) formed around the hydrogen atoms. The MEP values of atoms O1 and O2 are −0.050 and −0.017 a.u., respectively. Thus, atoms O1 and O2 are the most appropriate ones for electrophilic attacks while H atoms are more appropriate for nucleophilic attacks.

## Database survey   

Cholesterol and its esters take part significantly in many biological mechanisms, being important components for the manufacture of bile acids, steroid hormones and several fat-soluble vitamins. For the numerous experimental investigations, see: Faiman & Larsson, 1976 ▸; Goheen *et al.*, 1977 ▸; Bush *et al.*, 1980 ▸; Di Vizio *et al.*, 2008 ▸; Ikonen, 2008 ▸. For the first electron paramagnetic resonance (EPR) study of free radicals in X-ray-irradiated powdered cholesterol, hormones and vitamins, see: Rexroad & Gordy, 1959 ▸. For gamma-irradiated sterol groups studied at low temperatures, see: Sevilla *et al.*, 1986 ▸. For EPR and electron-nuclear double resonance (ENDOR) studies to elucidate the structure of free radicals formed in gamma-irradiated single crystals of selected steroids, see: Smaller & Matheson, 1958 ▸; Krzyminiewski, Hafez *et al.*, 1987 ▸; Krzyminiewski *et al.*, 1990 ▸; Szyczewski & Möbius, 1994 ▸; Szyczewski, 1996 ▸; Szyczewski *et al.*, 1998 ▸; Çalişkan *et al.*, 2004 ▸; Szyczewski *et al.*, 2005 ▸; Sayin *et al.*, 2011 ▸. For EPR studies of cholesteryl hepta­noate, see: Sayin *et al.*, 2013 ▸.

## Synthesis and crystallization   

The white fine crystalline powder of cholesteryl hepta­noate (C_34_H_58_O_2_) was purchased from Merck, and single crystals were grown by slow evaporation of a concentrated ethyl acetate solution.

## Refinement   

Crystal data, data collection and structure refinement details are summarized in Table 4 ▸. The C-bound H atoms were positioned geometrically, with C—H = 0.96, 0.97 and 0.98 Å for methyl, methyl­ene and methine H atoms, respectively, and constrained to ride on their parent atoms, with *U*_iso_(H) = *k* × *U*_eq_(C), where *k* = 1.5 for methyl H atoms and *k* = 1.2 for methyl­ene and methine H atoms.

## Supplementary Material

Crystal structure: contains datablock(s) I, global. DOI: 10.1107/S2056989021005661/mw2174sup1.cif

Structure factors: contains datablock(s) I. DOI: 10.1107/S2056989021005661/mw2174Isup2.hkl

CCDC reference: 2087356

Additional supporting information:  crystallographic information; 3D view; checkCIF report

## Figures and Tables

**Table 1:** Selected interatomic distances (Å)

O2⋯H8	2.43	H9*A*⋯H15	2.26
C9⋯H12*C*	2.78	H9*B*⋯H12*C*	2.30
C12⋯H19*A*	2.63	H12*A*⋯H19*A*	2.21
C13⋯H19*B*	2.79	H12*B*⋯H17	2.30
C17⋯H22*C*	2.78	H12*C*⋯H14*A*	2.37
C19⋯H22*C*	2.74	H13*A*⋯H19*B*	2.29
C19⋯H12*A*	2.73	H13*B*⋯H18	2.27
C22⋯H19*A*	2.77	H16*A*⋯H23	2.36
C22⋯H27	2.70	H17⋯H22*C*	2.26
C24⋯H22*B*	2.68	H19*A*⋯H22*C*	2.23
C25⋯H22*B*	2.71	H20*B*⋯H28*B*	2.17
C25⋯H29*A*	2.51	H22*B*⋯H24*B*	2.34
C28⋯H20*B*	2.78	H25*A*⋯H29*A*	2.32
C30⋯H28*A*	2.79	H28*A*⋯H30*A*	2.26
C30⋯H33*A*	2.75	H30*B*⋯H33*A*	2.33
H3*A*⋯H6*B*	2.31		

**Table 2:** Comparison of the selected (X-ray and DFT) geometric data (Å, °)

Bonds/angles	X-ray	B3LYP/6–31G(*d*)
O2—C7	1.196 (3)	1.21334
O1—C7	1.348 (3)	1.35309
O1—C8	1.458 (2)	1.45445
C7—C6	1.510 (3)	1.51813
C5—C6	1.516 (3)	1.53121
C5—C4	1.530 (3)	1.53654
C4—C3	1.512 (4)	1.53569
C3—C2	1.523 (3)	1.53425
C1—C2	1.510 (4)	1.53213
C8—C14	1.513 (3)	1.52760
C8—C9	1.518 (3)	1.52497
C10—C9	1.519 (3)	1.53951
C11—C12	1.545 (3)	1.54603
C11—C18	1.558 (3)	1.56904
C17—C18	1.544 (3)	1.55696
C22—C21	1.530 (3)	1.54490
C23—C21	1.538 (3)	1.55738
C24—C23	1.527 (3)	1.55738
C24—C25	1.538 (3)	1.55293
C26—C27	1.535 (3)	1.55117
C28—C27	1.528 (3)	1.53804
C29—C27	1.539 (3)	1.54887
C29—C30	1.525 (3)	1.53709
C31—C30	1.523 (3)	1.53617
C31—C32	1.524 (3)	1.54188
C33—C32	1.509 (4)	1.53652
C34—C32	1.518 (4)	1.53610
		
C1—C2—C3	113.9 (2)	113.26388
C3—C4—C5	115.3 (2)	114.95515
C5—C6—C7	113.7 (2)	112.96691
C6—C7—O1	110.5 (2)	110.59081
C7—O1—C8	117.58 (19)	117.36016
C9—C8—C14	110.85 (19)	111.83435
C10—C11—C13	108.31 (17)	107.22354
C16—C17—C18	110.06 (17)	111.14810
C18—C19—C20	113.82 (17)	113.68808
C20—C21—C23	106.26 (17)	106.65458
C23—C24—C25	103.79 (18)	103.66681
C26—C27—C29	110.60 (18)	110.09045
C29—C30—C31	112.0 (2)	112.44335
C31—C32—C33	113.3 (2)	112.54400
C31—C32—C34	110.2 (2)	110.56977
		
C1—C2—C3—C4	−177.7 (2)	179.78287
C6—C7—O1—C8	−179.5 (2)	179.67988
C9—C10—C11—C18	−166.45 (19)	164.70017
C16—C17—C23—C24	−57.6 (3)	−53.53645
C25—C26—C27—C29	56.7 (3)	58.14095
C29—C30—C31—C32	170.8 (2)	174.94079
C30—C31—C32—C33	58.8 (3)	63.49014
C30—C31—C32—C34	−176.9 (3)	−172.43112

**Table 3:** Calculated energies

Mol­ecular Energy (a.u.) (eV)	Compound (I)
Total Energy, *TE* (eV)	−40334.80
*E*_HOMO_ (eV)	−7.05
*E*_LUMO_ (eV)	−0.56
Gap, *ΔE* (eV)	6.49
Dipole moment, *μ* (Debye)	−4.07
Ionization potential, *I* (eV)	7.05
Electron affinity, *A*	0.56
Electronegativity, *χ*	4.06
Hardness, *η*	2.14
Electrophilicity index, *ω*	3.85
Softness, *σ*	0.23
Fraction of electron transferred, *ΔN*	0.49

**Table 4:** Experimental details

Crystal data	
Chemical formula	C_34_H_58_O_2_
*M*_r_	498.80
Crystal system, space group	Monoclinic, *P*2_1_
Temperature (K)	120
*a*, *b*, *c* (Å)	12.0622 (3), 9.2715 (2), 13.8140 (4)
β (°)	92.306 (2)
*V* (Å^3^)	1543.63 (7)
*Z*	2
Radiation type	Mo *K*α
μ (mm^−1^)	0.06
Crystal size (mm)	0.30 × 0.22 × 0.09

Data collection	
Diffractometer	Bruker APEXII QUAZAR three-circle diffractometer
No. of measured, independent and observed [*I* > 2σ(*I*)] reflections	15024, 6805, 6079
*R*_int_	0.041
(sin θ/λ)_max_ (Å^−1^)	0.649

Refinement	
*R*[*F*^2^ > 2σ(*F* ^2^)], *wR*(*F* ^2^), *S*	0.046, 0.118, 1.03
No. of reflections	6805
No. of parameters	331
No. of restraints	1
H-atom treatment	H-atom parameters constrained
Δρ_max_, Δρ_min_ (e Å^−3^)	0.24, −0.22
Absolute structure	Flack *x*determined using 2417 quotients [(*I* ^+^)−(*I* ^−^)]/[(*I* ^+^)+(*I* ^−^)] (Parsons *et al.*, 2013 ▸)
Absolute structure parameter	0.3 (7)

Computer programs: *APEX2* and *SAINT* (Bruker, 2012 ▸), *SHELXT* (Sheldrick, 2015*a* ▸), *SHELXL2018/3* (Sheldrick, 2015*b* ▸), *ORTEP-3* for Windows (Farrugia, 2012 ▸), *WinGX* publication routines (Farrugia, 2012 ▸) and *PLATON* (Spek, 2020 ▸).

## supplementary crystallographic information

### Crystal data

**Table:**

C_34_H_58_O_2_	*F*(000) = 556
*M**_r_* = 498.80	*D*_x_ = 1.073 Mg m^−^^3^
Monoclinic, *P*2_1_	Mo *K*α radiation, λ = 0.71073 Å
*a* = 12.0622 (3) Å	Cell parameters from 5761 reflections
*b* = 9.2715 (2) Å	θ = 2.2–27.3°
*c* = 13.8140 (4) Å	µ = 0.06 mm^−^^1^
β = 92.306 (2)°	*T* = 120 K
*V* = 1543.63 (7) Å^3^	Plate, colourless
*Z* = 2	0.30 × 0.22 × 0.09 mm

### Data collection

**Table:**

Bruker APEXII QUAZAR three-circle diffractometer	*R*_int_ = 0.041
Detector resolution: 8.3333 pixels mm^-1^	θ_max_ = 27.5°, θ_min_ = 1.5°
φ and ω scans	*h* = −15→15
15024 measured reflections	*k* = −12→12
6805 independent reflections	*l* = −17→17
6079 reflections with *I* > 2σ(*I*)	

### Refinement

**Table:**

Refinement on *F*^2^	Hydrogen site location: inferred from neighbouring sites
Least-squares matrix: full	H-atom parameters constrained
*R*[*F*^2^ > 2σ(*F*^2^)] = 0.046	*w* = 1/[σ^2^(*F*_o_^2^) + (0.0614*P*)^2^ + 0.1758*P*] where *P* = (*F*_o_^2^ + 2*F*_c_^2^)/3
*wR*(*F*^2^) = 0.118	(Δ/σ)_max_ < 0.001
*S* = 1.03	Δρ_max_ = 0.24 e Å^−^^3^
6805 reflections	Δρ_min_ = −0.22 e Å^−^^3^
331 parameters	Absolute structure: Flack *x*determined using 2417 quotients [(*I*^+^)-(*I*^-^)]/[(*I*^+^)+(*I*^-^)] (Parsons *et al.*, 2013)
1 restraint	Absolute structure parameter: 0.3 (7)

### Special details

**Table:**

Geometry. All esds (except the esd in the dihedral angle between two l.s. planes) are estimated using the full covariance matrix. The cell esds are taken into account individually in the estimation of esds in distances, angles and torsion angles; correlations between esds in cell parameters are only used when they are defined by crystal symmetry. An approximate (isotropic) treatment of cell esds is used for estimating esds involving l.s. planes.

### Fractional atomic coordinates and isotropic or equivalent isotropic displacement parameters (Å^2^)

**Table:**

	*x*	*y*	*z*	*U*_iso_*/*U*_eq_	
O1	0.88629 (14)	0.64631 (18)	0.15841 (13)	0.0314 (4)	
O2	0.91179 (18)	0.8775 (2)	0.11617 (18)	0.0518 (6)	
C1	1.4569 (2)	0.5375 (3)	−0.03304 (19)	0.0381 (6)	
H1A	1.504609	0.496801	−0.079854	0.057*	
H1B	1.431548	0.462591	0.008619	0.057*	
H1C	1.497197	0.608290	0.004961	0.057*	
C2	1.3584 (2)	0.6081 (3)	−0.08471 (17)	0.0316 (5)	
H2A	1.320002	0.536527	−0.124748	0.038*	
H2B	1.385067	0.682562	−0.127298	0.038*	
C3	1.2763 (2)	0.6752 (3)	−0.01697 (17)	0.0330 (6)	
H3A	1.251610	0.601733	0.027306	0.040*	
H3B	1.313740	0.749700	0.021227	0.040*	
C4	1.1761 (2)	0.7401 (3)	−0.06989 (18)	0.0308 (5)	
H4A	1.140592	0.665786	−0.109617	0.037*	
H4B	1.201347	0.814656	−0.113085	0.037*	
C5	1.0894 (2)	0.8056 (3)	−0.00503 (19)	0.0304 (5)	
H5A	1.124902	0.877278	0.036917	0.036*	
H5B	1.033294	0.854229	−0.045343	0.036*	
C6	1.0337 (2)	0.6940 (3)	0.0568 (2)	0.0367 (6)	
H6A	1.006225	0.616313	0.015417	0.044*	
H6B	1.088515	0.653667	0.102419	0.044*	
C7	0.9385 (2)	0.7534 (3)	0.1125 (2)	0.0335 (6)	
C8	0.79138 (19)	0.6851 (3)	0.21509 (17)	0.0273 (5)	
H8	0.804295	0.779292	0.245756	0.033*	
C9	0.78323 (19)	0.5697 (3)	0.29241 (17)	0.0261 (5)	
H9A	0.848991	0.572960	0.335142	0.031*	
H9B	0.780098	0.475481	0.261901	0.031*	
C10	0.68089 (18)	0.5910 (2)	0.35138 (16)	0.0220 (4)	
C11	0.57079 (18)	0.6073 (2)	0.29501 (15)	0.0205 (4)	
C12	0.5379 (2)	0.4598 (3)	0.25045 (17)	0.0267 (5)	
H12A	0.475360	0.471934	0.206043	0.040*	
H12B	0.518810	0.394491	0.301058	0.040*	
H12C	0.599259	0.421289	0.216544	0.040*	
C13	0.58615 (19)	0.7181 (3)	0.21267 (16)	0.0247 (5)	
H13A	0.519863	0.717802	0.170516	0.030*	
H13B	0.593196	0.813491	0.241059	0.030*	
C14	0.68645 (19)	0.6904 (3)	0.15136 (17)	0.0278 (5)	
H14A	0.676936	0.599652	0.117096	0.033*	
H14B	0.692363	0.766606	0.103719	0.033*	
C15	0.69011 (18)	0.5926 (2)	0.44767 (16)	0.0242 (5)	
H15	0.760872	0.583827	0.476350	0.029*	
C16	0.59494 (18)	0.6076 (3)	0.51323 (15)	0.0249 (5)	
H16A	0.596925	0.703036	0.542050	0.030*	
H16B	0.603350	0.537672	0.565224	0.030*	
C17	0.48277 (18)	0.5853 (2)	0.46075 (15)	0.0205 (4)	
H17	0.471506	0.481913	0.449347	0.025*	
C18	0.47990 (17)	0.6643 (2)	0.36225 (15)	0.0195 (4)	
H18	0.498642	0.765154	0.376379	0.023*	
C19	0.36285 (18)	0.6654 (3)	0.31454 (15)	0.0241 (5)	
H19A	0.344502	0.568507	0.292694	0.029*	
H19B	0.362694	0.727231	0.257918	0.029*	
C20	0.27255 (18)	0.7178 (3)	0.38188 (16)	0.0237 (5)	
H20A	0.285173	0.818564	0.397559	0.028*	
H20B	0.200528	0.709896	0.348451	0.028*	
C21	0.27233 (17)	0.6294 (2)	0.47595 (15)	0.0199 (4)	
C22	0.2384 (2)	0.4731 (2)	0.45499 (18)	0.0269 (5)	
H22A	0.166143	0.471404	0.423254	0.040*	
H22B	0.236660	0.420533	0.514769	0.040*	
H22C	0.291140	0.429430	0.413806	0.040*	
C23	0.39003 (17)	0.6415 (2)	0.52227 (14)	0.0196 (4)	
H23	0.404155	0.744866	0.531215	0.024*	
C24	0.3794 (2)	0.5788 (3)	0.62369 (16)	0.0272 (5)	
H24A	0.436235	0.617339	0.668192	0.033*	
H24B	0.385290	0.474515	0.622739	0.033*	
C25	0.26318 (19)	0.6266 (3)	0.65246 (16)	0.0266 (5)	
H25A	0.268795	0.698477	0.703457	0.032*	
H25B	0.221731	0.544824	0.675685	0.032*	
C26	0.20397 (17)	0.6915 (2)	0.55966 (15)	0.0210 (4)	
H26	0.216305	0.795963	0.561267	0.025*	
C27	0.07807 (18)	0.6667 (3)	0.55763 (15)	0.0251 (5)	
H27	0.065026	0.562329	0.557622	0.030*	
C28	0.0200 (2)	0.7293 (3)	0.46669 (18)	0.0342 (6)	
H28A	−0.058932	0.724501	0.472814	0.051*	
H28B	0.040410	0.674760	0.411099	0.051*	
H28C	0.041967	0.828039	0.459043	0.051*	
C29	0.02759 (19)	0.7298 (3)	0.64897 (17)	0.0295 (5)	
H29A	0.076544	0.707596	0.704403	0.035*	
H29B	0.024675	0.833916	0.642601	0.035*	
C30	−0.08842 (19)	0.6749 (3)	0.66924 (16)	0.0274 (5)	
H30A	−0.139634	0.705361	0.617273	0.033*	
H30B	−0.087729	0.570343	0.670378	0.033*	
C31	−0.1289 (2)	0.7309 (3)	0.76532 (18)	0.0355 (6)	
H31A	−0.117039	0.834280	0.767913	0.043*	
H31B	−0.083762	0.687938	0.817400	0.043*	
C32	−0.2505 (2)	0.7007 (3)	0.78366 (17)	0.0302 (5)	
H32	−0.295137	0.747125	0.731650	0.036*	
C33	−0.2788 (3)	0.5422 (3)	0.7824 (2)	0.0433 (7)	
H33A	−0.262037	0.501912	0.720684	0.065*	
H33B	−0.356401	0.530157	0.793139	0.065*	
H33C	−0.236003	0.493674	0.832650	0.065*	
C34	−0.2821 (3)	0.7685 (4)	0.8787 (2)	0.0544 (9)	
H34A	−0.266938	0.870126	0.877199	0.082*	
H34B	−0.239742	0.724853	0.931153	0.082*	
H34C	−0.359790	0.753511	0.887713	0.082*	

### Atomic displacement parameters (Å^2^)

**Table:**

	*U*^11^	*U*^22^	*U*^33^	*U*^12^	*U*^13^	*U*^23^
O1	0.0290 (9)	0.0269 (9)	0.0393 (10)	0.0008 (7)	0.0154 (7)	−0.0006 (7)
O2	0.0526 (13)	0.0282 (10)	0.0772 (16)	0.0055 (9)	0.0329 (11)	0.0059 (10)
C1	0.0320 (14)	0.0523 (17)	0.0305 (13)	−0.0027 (12)	0.0073 (11)	0.0017 (12)
C2	0.0344 (13)	0.0339 (13)	0.0269 (12)	−0.0017 (11)	0.0082 (10)	0.0022 (10)
C3	0.0293 (12)	0.0445 (15)	0.0253 (11)	−0.0018 (11)	0.0048 (9)	0.0024 (11)
C4	0.0330 (13)	0.0312 (13)	0.0287 (12)	−0.0034 (10)	0.0087 (10)	0.0065 (10)
C5	0.0315 (13)	0.0260 (12)	0.0339 (13)	−0.0021 (10)	0.0053 (10)	0.0026 (10)
C6	0.0329 (13)	0.0294 (13)	0.0492 (15)	0.0020 (11)	0.0183 (11)	0.0053 (12)
C7	0.0314 (13)	0.0293 (13)	0.0406 (14)	−0.0007 (10)	0.0104 (11)	0.0020 (11)
C8	0.0262 (11)	0.0255 (11)	0.0309 (12)	0.0001 (10)	0.0114 (9)	−0.0057 (10)
C9	0.0222 (11)	0.0281 (12)	0.0281 (11)	0.0011 (9)	0.0028 (9)	−0.0035 (9)
C10	0.0224 (11)	0.0172 (10)	0.0265 (11)	0.0008 (8)	0.0039 (8)	−0.0032 (8)
C11	0.0219 (10)	0.0204 (11)	0.0195 (10)	−0.0006 (8)	0.0029 (8)	−0.0028 (8)
C12	0.0285 (12)	0.0244 (11)	0.0275 (12)	−0.0006 (9)	0.0035 (9)	−0.0071 (9)
C13	0.0279 (11)	0.0252 (12)	0.0215 (10)	0.0042 (9)	0.0057 (9)	−0.0004 (9)
C14	0.0328 (12)	0.0281 (12)	0.0231 (11)	0.0027 (10)	0.0097 (9)	−0.0008 (9)
C15	0.0196 (10)	0.0251 (11)	0.0279 (11)	0.0010 (9)	−0.0007 (8)	−0.0013 (9)
C16	0.0249 (11)	0.0302 (12)	0.0197 (10)	0.0039 (10)	0.0012 (8)	−0.0003 (9)
C17	0.0223 (10)	0.0193 (10)	0.0201 (10)	0.0020 (8)	0.0031 (8)	−0.0001 (8)
C18	0.0220 (10)	0.0184 (10)	0.0183 (10)	0.0003 (8)	0.0030 (8)	−0.0018 (8)
C19	0.0237 (11)	0.0301 (12)	0.0185 (10)	0.0015 (9)	0.0020 (8)	0.0010 (9)
C20	0.0214 (10)	0.0275 (12)	0.0222 (10)	0.0025 (9)	0.0014 (8)	0.0010 (9)
C21	0.0201 (10)	0.0202 (10)	0.0196 (10)	0.0004 (8)	0.0018 (8)	−0.0010 (8)
C22	0.0282 (12)	0.0242 (12)	0.0289 (12)	−0.0014 (9)	0.0077 (9)	−0.0052 (9)
C23	0.0218 (10)	0.0189 (10)	0.0182 (10)	0.0024 (8)	0.0021 (8)	0.0001 (8)
C24	0.0290 (12)	0.0309 (12)	0.0218 (11)	0.0071 (10)	0.0040 (9)	0.0045 (9)
C25	0.0296 (12)	0.0307 (12)	0.0198 (10)	0.0046 (10)	0.0046 (9)	0.0023 (9)
C26	0.0231 (10)	0.0205 (10)	0.0197 (10)	0.0017 (9)	0.0021 (8)	−0.0011 (8)
C27	0.0236 (11)	0.0284 (12)	0.0237 (11)	−0.0004 (9)	0.0054 (8)	−0.0043 (9)
C28	0.0238 (12)	0.0484 (16)	0.0306 (13)	0.0023 (11)	0.0035 (9)	−0.0017 (11)
C29	0.0250 (11)	0.0375 (14)	0.0265 (12)	0.0007 (10)	0.0060 (9)	−0.0080 (10)
C30	0.0272 (11)	0.0301 (12)	0.0252 (11)	0.0006 (10)	0.0061 (9)	−0.0047 (10)
C31	0.0302 (13)	0.0496 (16)	0.0273 (12)	−0.0016 (11)	0.0078 (10)	−0.0125 (11)
C32	0.0317 (12)	0.0334 (13)	0.0262 (11)	0.0029 (10)	0.0095 (9)	0.0004 (10)
C33	0.0514 (17)	0.0420 (16)	0.0376 (15)	−0.0039 (13)	0.0157 (13)	−0.0029 (12)
C34	0.0498 (18)	0.061 (2)	0.0543 (19)	−0.0098 (15)	0.0281 (15)	−0.0249 (16)

### Geometric parameters (Å, º)

**Table:**

O1—C7	1.348 (3)	C18—C19	1.534 (3)
O1—C8	1.458 (2)	C18—H18	0.9800
O2—C7	1.196 (3)	C19—C20	1.539 (3)
C1—C2	1.510 (4)	C19—H19A	0.9700
C1—H1A	0.9600	C19—H19B	0.9700
C1—H1B	0.9600	C20—C21	1.536 (3)
C1—H1C	0.9600	C20—H20A	0.9700
C2—C3	1.523 (3)	C20—H20B	0.9700
C2—H2A	0.9700	C21—C22	1.530 (3)
C2—H2B	0.9700	C21—C23	1.538 (3)
C3—C4	1.512 (4)	C21—C26	1.557 (3)
C3—H3A	0.9700	C22—H22A	0.9600
C3—H3B	0.9700	C22—H22B	0.9600
C4—C5	1.530 (3)	C22—H22C	0.9600
C4—H4A	0.9700	C23—C24	1.527 (3)
C4—H4B	0.9700	C23—H23	0.9800
C5—C6	1.516 (3)	C24—C25	1.538 (3)
C5—H5A	0.9700	C24—H24A	0.9700
C5—H5B	0.9700	C24—H24B	0.9700
C6—C7	1.510 (3)	C25—C26	1.563 (3)
C6—H6A	0.9700	C25—H25A	0.9700
C6—H6B	0.9700	C25—H25B	0.9700
C8—C14	1.513 (3)	C26—C27	1.535 (3)
C8—C9	1.518 (3)	C26—H26	0.9800
C8—H8	0.9800	C27—C28	1.528 (3)
C9—C10	1.519 (3)	C27—C29	1.539 (3)
C9—H9A	0.9700	C27—H27	0.9800
C9—H9B	0.9700	C28—H28A	0.9600
C10—C15	1.330 (3)	C28—H28B	0.9600
C10—C11	1.520 (3)	C28—H28C	0.9600
C11—C12	1.545 (3)	C29—C30	1.525 (3)
C11—C13	1.549 (3)	C29—H29A	0.9700
C11—C18	1.558 (3)	C29—H29B	0.9700
C12—H12A	0.9600	C30—C31	1.523 (3)
C12—H12B	0.9600	C30—H30A	0.9700
C12—H12C	0.9600	C30—H30B	0.9700
C13—C14	1.526 (3)	C31—C32	1.524 (3)
C13—H13A	0.9700	C31—H31A	0.9700
C13—H13B	0.9700	C31—H31B	0.9700
C14—H14A	0.9700	C32—C33	1.509 (4)
C14—H14B	0.9700	C32—C34	1.518 (4)
C15—C16	1.497 (3)	C32—H32	0.9800
C15—H15	0.9300	C33—H33A	0.9600
C16—C17	1.523 (3)	C33—H33B	0.9600
C16—H16A	0.9700	C33—H33C	0.9600
C16—H16B	0.9700	C34—H34A	0.9600
C17—C23	1.524 (3)	C34—H34B	0.9600
C17—C18	1.544 (3)	C34—H34C	0.9600
C17—H17	0.9800		
			
O2···C14	3.277 (2)	H4B···H9B^i^	2.56
O2···H5A	2.83	H5A···H33C^vii^	2.45
O2···H5B	2.73	H5B···H6A^i^	2.51
O2···H8	2.43	H8···H13B	2.56
O2···H14B	2.84	H9A···H15	2.26
O2···H4A^i^	2.75	H9A···H28A^v^	2.58
C20···C28	3.309 (2)	H9B···H12C	2.30
C22···C28	3.556 (2)	H9B···H14A	2.58
C3···H6B	2.86	H12A···H13A	2.40
C6···H3A	2.81	H12A···H19A	2.21
C7···H14B	2.97	H12B···H17	2.30
C9···H12C	2.78	H12C···H14A	2.37
C12···H17	2.90	H13A···H19B	2.29
C12···H9B	2.92	H13B···H18	2.27
C12···H14A	2.85	H13B···H24B^vii^	2.41
C12···H19A	2.63	H14B···H34B^viii^	2.58
C13···H19B	2.79	H15···H28A^v^	2.54
C14···H12C	2.87	H15···H30A^v^	2.51
C15···H18	2.95	H16A···H18	2.60
C15···H26^ii^	2.98	H16A···H23	2.36
C16···H24A	2.93	H16A···H22C^vii^	2.56
C17···H12B	2.87	H16B···H20A^ii^	2.48
C17···H22C	2.78	H17···H22C	2.26
C19···H22C	2.74	H18···H23	2.47
C19···H12A	2.73	H18···H24B^vii^	2.39
C19···H13A	2.84	H19A···H22C	2.23
C20···H28B	2.87	H20A···H23	2.39
C21···H28B	2.93	H20A···H26	2.45
C22···H19A	2.77	H20A···H33A^ix^	2.37
C22···H24B	2.86	H20B···H22A	2.48
C22···H30A^iii^	2.91	H20B···H28B	2.17
C22···H27	2.70	H22A···H27	2.41
C22···H17	2.82	H22A···H28B	2.42
C24···H16B	2.88	H22A···H30A^iii^	2.55
C24···H22B	2.68	H22B···H24B	2.34
C25···H22B	2.71	H22B···H25B	2.52
C25···H29A	2.51	H22B···H27	2.54
C27···H22A	2.83	H23···H26	2.37
C28···H20B	2.78	H25A···H29A	2.32
C28···H30A	2.90	H25B···H27	2.45
C29···H25B	2.91	H25B···H29A	2.36
C30···H28A	2.79	H26···H28C	2.50
C30···H33A	2.75	H27···H30B	2.46
C33···H30B	2.84	H27···H28C^iii^	2.53
H1A···H33B^iv^	2.49	H28A···H30A	2.26
H1B···H3A	2.55	H28C···H29B	2.55
H1B···H34C^ii^	2.58	H29A···H31B	2.54
H1C···H3B	2.59	H29B···H28C	2.55
H1C···H13A^v^	2.51	H29B···H31A	2.48
H2A···H4A	2.49	H30A···H32	2.53
H2A···H14B^vi^	2.52	H30B···H33A	2.33
H2B···H4B	2.55	H31A···H34A	2.42
H2B···H12C^i^	2.54	H31B···H33C	2.59
H3A···H6B	2.31	H31B···H34B	2.52
H3A···H34A^ii^	2.52	H33B···H34C	2.45
H3B···H5A	2.58	H33C···H34B	2.54
H4A···H6A	2.46		
			
C7—O1—C8	117.58 (19)	C17—C18—H18	106.3
C2—C1—H1A	109.5	C11—C18—H18	106.3
C2—C1—H1B	109.5	C18—C19—C20	113.82 (17)
H1A—C1—H1B	109.5	C18—C19—H19A	108.8
C2—C1—H1C	109.5	C20—C19—H19A	108.8
H1A—C1—H1C	109.5	C18—C19—H19B	108.8
H1B—C1—H1C	109.5	C20—C19—H19B	108.8
C1—C2—C3	113.9 (2)	H19A—C19—H19B	107.7
C1—C2—H2A	108.8	C21—C20—C19	111.60 (18)
C3—C2—H2A	108.8	C21—C20—H20A	109.3
C1—C2—H2B	108.8	C19—C20—H20A	109.3
C3—C2—H2B	108.8	C21—C20—H20B	109.3
H2A—C2—H2B	107.7	C19—C20—H20B	109.3
C4—C3—C2	113.1 (2)	H20A—C20—H20B	108.0
C4—C3—H3A	109.0	C22—C21—C20	110.77 (18)
C2—C3—H3A	109.0	C22—C21—C23	112.56 (18)
C4—C3—H3B	109.0	C20—C21—C23	106.26 (17)
C2—C3—H3B	109.0	C22—C21—C26	110.19 (18)
H3A—C3—H3B	107.8	C20—C21—C26	116.73 (18)
C3—C4—C5	115.3 (2)	C23—C21—C26	99.87 (16)
C3—C4—H4A	108.5	C21—C22—H22A	109.5
C5—C4—H4A	108.5	C21—C22—H22B	109.5
C3—C4—H4B	108.5	H22A—C22—H22B	109.5
C5—C4—H4B	108.5	C21—C22—H22C	109.5
H4A—C4—H4B	107.5	H22A—C22—H22C	109.5
C6—C5—C4	112.9 (2)	H22B—C22—H22C	109.5
C6—C5—H5A	109.0	C17—C23—C24	118.16 (18)
C4—C5—H5A	109.0	C17—C23—C21	115.40 (17)
C6—C5—H5B	109.0	C24—C23—C21	104.12 (17)
C4—C5—H5B	109.0	C17—C23—H23	106.1
H5A—C5—H5B	107.8	C24—C23—H23	106.1
C7—C6—C5	113.7 (2)	C21—C23—H23	106.1
C7—C6—H6A	108.8	C23—C24—C25	103.79 (18)
C5—C6—H6A	108.8	C23—C24—H24A	111.0
C7—C6—H6B	108.8	C25—C24—H24A	111.0
C5—C6—H6B	108.8	C23—C24—H24B	111.0
H6A—C6—H6B	107.7	C25—C24—H24B	111.0
O2—C7—O1	123.8 (2)	H24A—C24—H24B	109.0
O2—C7—C6	125.7 (2)	C24—C25—C26	106.87 (17)
O1—C7—C6	110.5 (2)	C24—C25—H25A	110.3
O1—C8—C14	110.61 (18)	C26—C25—H25A	110.3
O1—C8—C9	106.15 (18)	C24—C25—H25B	110.3
C14—C8—C9	110.85 (19)	C26—C25—H25B	110.3
O1—C8—H8	109.7	H25A—C25—H25B	108.6
C14—C8—H8	109.7	C27—C26—C21	118.94 (17)
C9—C8—H8	109.7	C27—C26—C25	112.11 (18)
C8—C9—C10	111.26 (19)	C21—C26—C25	103.22 (17)
C8—C9—H9A	109.4	C27—C26—H26	107.3
C10—C9—H9A	109.4	C21—C26—H26	107.3
C8—C9—H9B	109.4	C25—C26—H26	107.3
C10—C9—H9B	109.4	C28—C27—C26	112.24 (18)
H9A—C9—H9B	108.0	C28—C27—C29	110.25 (19)
C15—C10—C9	120.0 (2)	C26—C27—C29	110.60 (18)
C15—C10—C11	123.19 (19)	C28—C27—H27	107.9
C9—C10—C11	116.77 (18)	C26—C27—H27	107.9
C10—C11—C12	108.74 (18)	C29—C27—H27	107.9
C10—C11—C13	108.31 (17)	C27—C28—H28A	109.5
C12—C11—C13	109.31 (18)	C27—C28—H28B	109.5
C10—C11—C18	110.47 (17)	H28A—C28—H28B	109.5
C12—C11—C18	111.26 (18)	C27—C28—H28C	109.5
C13—C11—C18	108.70 (17)	H28A—C28—H28C	109.5
C11—C12—H12A	109.5	H28B—C28—H28C	109.5
C11—C12—H12B	109.5	C30—C29—C27	114.8 (2)
H12A—C12—H12B	109.5	C30—C29—H29A	108.6
C11—C12—H12C	109.5	C27—C29—H29A	108.6
H12A—C12—H12C	109.5	C30—C29—H29B	108.6
H12B—C12—H12C	109.5	C27—C29—H29B	108.6
C14—C13—C11	114.64 (18)	H29A—C29—H29B	107.5
C14—C13—H13A	108.6	C31—C30—C29	112.0 (2)
C11—C13—H13A	108.6	C31—C30—H30A	109.2
C14—C13—H13B	108.6	C29—C30—H30A	109.2
C11—C13—H13B	108.6	C31—C30—H30B	109.2
H13A—C13—H13B	107.6	C29—C30—H30B	109.2
C8—C14—C13	110.22 (18)	H30A—C30—H30B	107.9
C8—C14—H14A	109.6	C30—C31—C32	115.3 (2)
C13—C14—H14A	109.6	C30—C31—H31A	108.5
C8—C14—H14B	109.6	C32—C31—H31A	108.5
C13—C14—H14B	109.6	C30—C31—H31B	108.5
H14A—C14—H14B	108.1	C32—C31—H31B	108.5
C10—C15—C16	124.8 (2)	H31A—C31—H31B	107.5
C10—C15—H15	117.6	C33—C32—C34	110.4 (2)
C16—C15—H15	117.6	C33—C32—C31	113.3 (2)
C15—C16—C17	112.80 (18)	C34—C32—C31	110.2 (2)
C15—C16—H16A	109.0	C33—C32—H32	107.6
C17—C16—H16A	109.0	C34—C32—H32	107.6
C15—C16—H16B	109.0	C31—C32—H32	107.6
C17—C16—H16B	109.0	C32—C33—H33A	109.5
H16A—C16—H16B	107.8	C32—C33—H33B	109.5
C16—C17—C23	110.24 (17)	H33A—C33—H33B	109.5
C16—C17—C18	110.06 (17)	C32—C33—H33C	109.5
C23—C17—C18	109.77 (17)	H33A—C33—H33C	109.5
C16—C17—H17	108.9	H33B—C33—H33C	109.5
C23—C17—H17	108.9	C32—C34—H34A	109.5
C18—C17—H17	108.9	C32—C34—H34B	109.5
C19—C18—C17	111.67 (17)	H34A—C34—H34B	109.5
C19—C18—C11	113.81 (17)	C32—C34—H34C	109.5
C17—C18—C11	111.91 (17)	H34A—C34—H34C	109.5
C19—C18—H18	106.3	H34B—C34—H34C	109.5
			
C1—C2—C3—C4	−177.7 (2)	C12—C11—C18—C17	76.1 (2)
C2—C3—C4—C5	178.5 (2)	C13—C11—C18—C17	−163.47 (18)
C3—C4—C5—C6	−65.4 (3)	C17—C18—C19—C20	50.5 (2)
C4—C5—C6—C7	−173.1 (2)	C11—C18—C19—C20	178.42 (19)
C8—O1—C7—O2	0.5 (4)	C18—C19—C20—C21	−55.4 (3)
C8—O1—C7—C6	−179.5 (2)	C19—C20—C21—C22	−66.0 (2)
C5—C6—C7—O2	−5.8 (4)	C19—C20—C21—C23	56.5 (2)
C5—C6—C7—O1	174.2 (2)	C19—C20—C21—C26	166.82 (18)
C7—O1—C8—C14	85.5 (3)	C16—C17—C23—C24	−57.6 (3)
C7—O1—C8—C9	−154.2 (2)	C18—C17—C23—C24	−179.04 (19)
O1—C8—C9—C10	−175.13 (18)	C16—C17—C23—C21	178.27 (18)
C14—C8—C9—C10	−55.0 (2)	C18—C17—C23—C21	56.9 (2)
C8—C9—C10—C15	−129.1 (2)	C22—C21—C23—C17	61.6 (2)
C8—C9—C10—C11	51.9 (3)	C20—C21—C23—C17	−59.8 (2)
C15—C10—C11—C12	−107.8 (2)	C26—C21—C23—C17	178.40 (18)
C9—C10—C11—C12	71.2 (2)	C22—C21—C23—C24	−69.6 (2)
C15—C10—C11—C13	133.5 (2)	C20—C21—C23—C24	168.99 (18)
C9—C10—C11—C13	−47.5 (2)	C26—C21—C23—C24	47.2 (2)
C15—C10—C11—C18	14.6 (3)	C17—C23—C24—C25	−165.15 (19)
C9—C10—C11—C18	−166.45 (19)	C21—C23—C24—C25	−35.6 (2)
C10—C11—C13—C14	49.6 (2)	C23—C24—C25—C26	9.7 (3)
C12—C11—C13—C14	−68.7 (2)	C22—C21—C26—C27	−46.1 (3)
C18—C11—C13—C14	169.67 (18)	C20—C21—C26—C27	81.3 (3)
O1—C8—C14—C13	175.0 (2)	C23—C21—C26—C27	−164.72 (19)
C9—C8—C14—C13	57.6 (2)	C22—C21—C26—C25	78.7 (2)
C11—C13—C14—C8	−56.5 (3)	C20—C21—C26—C25	−153.80 (19)
C9—C10—C15—C16	−177.8 (2)	C23—C21—C26—C25	−39.9 (2)
C11—C10—C15—C16	1.2 (4)	C24—C25—C26—C27	148.31 (19)
C10—C15—C16—C17	13.7 (3)	C24—C25—C26—C21	19.1 (2)
C15—C16—C17—C23	−164.41 (19)	C21—C26—C27—C28	−59.3 (3)
C15—C16—C17—C18	−43.2 (2)	C25—C26—C27—C28	−179.7 (2)
C16—C17—C18—C19	−170.74 (18)	C21—C26—C27—C29	177.2 (2)
C23—C17—C18—C19	−49.2 (2)	C25—C26—C27—C29	56.7 (3)
C16—C17—C18—C11	60.4 (2)	C28—C27—C29—C30	72.1 (3)
C23—C17—C18—C11	−178.14 (17)	C26—C27—C29—C30	−163.2 (2)
C10—C11—C18—C19	−172.54 (18)	C27—C29—C30—C31	174.7 (2)
C12—C11—C18—C19	−51.7 (2)	C29—C30—C31—C32	170.8 (2)
C13—C11—C18—C19	68.8 (2)	C30—C31—C32—C33	58.8 (3)
C10—C11—C18—C17	−44.8 (2)	C30—C31—C32—C34	−176.9 (3)

Symmetry codes: (i) −*x*+2, *y*+1/2, −*z*; (ii) −*x*+1, *y*−1/2, −*z*+1; (iii) −*x*, *y*−1/2, −*z*+1; (iv) *x*+2, *y*, *z*−1; (v) *x*+1, *y*, *z*; (vi) −*x*+2, *y*−1/2, −*z*; (vii) −*x*+1, *y*+1/2, −*z*+1; (viii) *x*+1, *y*, *z*−1; (ix) −*x*, *y*+1/2, −*z*+1.
